# Effectiveness of a Recertification Vision Screening Training Module for Preschool Teachers

**DOI:** 10.21315/mjms2023.30.6.14

**Published:** 2023-12-19

**Authors:** Xuan Li Tan, Rokiah Omar, Victor Feizal Knight

**Affiliations:** 1 Faculty of Optometry and Vision Sciences, SEGi University, Selangor, Malaysia; 2 Optometry and Vision Sciences Programme, School of Healthcare Sciences, Faculty of Health Sciences, Universiti Kebangsaan Malaysia, Kuala Lumpur, Malaysia; 3 Faculty of Medicine and Defence Health, Universiti Pertahanan Malaysia, Kuala Lumpur, Malaysia

**Keywords:** preschool teacher, vision screening, vision impairment, lay screener, revalidation

## Abstract

**Background:**

Certified training must be provided for lay vision screeners prior to their conduct of a vision screening programme. However, the effectiveness of trained lay screeners does deteriorate over time. This study aims to evaluate the effectiveness of a recertification vision screening training module using the KieVision^™^ Preschool Vision Screening Kit for preschool teachers in Malaysia.

**Methods:**

This was a randomised control trial. Fifty-nine preschool teachers previously enrolled in a Knowledge Transfer Programme were divided into a Study Group (*n* = 31) to receive recertification training and a Control Group (*n* = 28) to attend briefing sessions. Subjects was then asked to perform vision screening on 15 preschool children aged 4 years old–6 years old at their respective premises, then verified by optometrists after 2 weeks from the initial screening on the same children.

**Results:**

A total of 894 children were screened, with the Study Group and Control Group screened 49.7% and 50.3%, respectively. There was higher validity in vision screening findings from the Study Group (sensitivity = 66.7%, positive predictive value (PPV) = 61.5%) compared to the Control Group (sensitivity = 36.0 %, PPV = 40.9%).

**Conclusion:**

Teachers who received recertification training were more competent in detecting children’s vision impairment using KieVision^™^ Preschool Vision Screening Kit. Thus, timely recertification training should be emphasised to ensure sustainable consistency and reliability of vision screening programmes conducted by lay vision screeners.

## Introduction

Visual development is crucial for children before age 7 years old as any uncorrected visual problem they suffer may impair their visual maturation in its critical period (1). Thus, vision screening is advisable for these children in their early preschool years. Currently, vision screening is often conducted by eye care professionals such as optometrists, orthoptists, ophthalmologists and nurses (2–5). However, a study showed that lay screeners specifically trained to conduct vision screening could produce valid results that meet the local community’s needs (6). The American National Center for Children’s Vision and Eye Health recommends that all vision screeners should attend a comprehensive training programme, preferably in conjunction with standardised training modules and uniform certification in the visual screening test methods to be used together with continuous education and formalised certification every 3–5 years (7).

This demonstrates that the international eye care community recognises the need to train screeners in screening vision for children to obtain the highest results from screening tests. Most studies described some training for lay screeners before they conduct vision screening. Several vision screening programmes are also developed to train lay screeners who may be teachers (5, 8–11) or parents (12) to conduct children’s vision screening. However, the literature has only minimal discussions on the appropriate duration, training coverage and content, which would be needed to produce the same level of effectiveness of vision screening.

In Malaysia, Omar et al. (6) pioneered the certification module for preschool vision screening, in which preschool teachers were involved as vision screeners. In this study, preschool teachers were recruited as screeners and required to attend a comprehensive participative training that includes theoretical and practical sessions on conducting vision screening. The teachers who completed the said course become certified screeners eligible to perform vision screening tests on preschool children using the KieVision^™^ Preschool Vision Screening Kit. The trained teachers could perform vision screening effectively on children as young as 4 years old using the training provided. There was no statistical difference (*P* > 0.05) when comparing the vision screening outcome between optometrists and trained preschool teachers. These trained teachers produced good validity in their vision screening results, where sensitivity and specificity of 79% and 95%, respectively, were determined.

Omar et al. (13) also reported a significantly higher knowledge level (*P* < 0.001) when the results of the written theoretical exam on the conduct of preschool vision screening were compared between trained teachers (73.2 ± 11.7) and the Control Group who received short briefing (56.2 ± 13.1) (13). However, this original module was developed without consideration for any regular competency testing after the initial training. Likely, the trained vision screeners who only underwent the initial training would eventually forget some or even all the specific details of screening techniques and referral criteria taught. Therefore, a method must ensure the maintenance of the competence of vision screeners. To do this, establishing a system that recertifies competence is needed. It is proposed that this is done by creating a recertification module for preschool vision screening to ensure the long-term continuance of both consistency and effectiveness of the vision screening programme.

Although current literature shows that teachers have been widely trained to function as vision screeners, the skill retention of these trained teachers after latency beyond initial training is unknown. Recently, Tan et al. (14) observed that trained preschool teachers showed a significant deterioration (about 18%) of their knowledge regarding preschool vision screening and vision impairments following a 3-years duration after initial training; the assessment used the same set of theory exam questions. It was suggested that there must be a timely refresher course to ensure the reliability of the results of the vision screening programme conducted by these trained teachers. Thus, this study evaluates the effectiveness of the recertification vision screening training module using the KieVision^™^ Preschool Vision Screening Kit for preschool teachers in Malaysia.

## Methods

This was a randomised controlled trial. The targeted population was preschool teachers in Malaysia. The study population comprises the Department of Community Development (KEMAS) TABIKA teachers in Selangor. These are preschool teachers employed by the KEMAS, Selangor State, Ministry of Rural and Regional Development Malaysia. The sampling method used was stratified random sampling. The criteria include active preschool teachers who had participated in the Knowledge Transfer Programme conducted in 2013. This pool of subjects was created in 2013 when 180 KEMAS preschool teachers were recruited for this programme. They attended the certification training to perform vision screening using the KieVision^™^ Preschool Vision Screening Kit, comprising theoretical and practical sessions. Those who completed the training and skill assessment were certified as preschool vision screeners (6).

This study required 30 subjects (*n*) in the Study and Control Groups, respectively, as calculated from the formula of Allen (15), which referenced comparisons between two independent means (15). This study’s vision screening programme involved four test constructs: i) observation of the child’s behaviour, ii) observation of the child’s external eye, iii) Hirschberg’s test and iv) distant visual acuity (VA) test. The VA value has often been used as the standard deviation value because it is the most important component among the vision screening tests performed. Thus, the VA value was deemed the most suitable for this formula. In this study, it was intended to detect if there was a difference of 0.1 logMAR between the Study Group and Control Group with 80% study power at 5% significance level. Assuming a 10% dropout rate, the sample size for the Study Group and the Control Group was each determined to be 33 subjects. Therefore, this study involved 66 preschool teachers randomly assigned into two groups using a random number table: Study Group (*n* = 33) and Control Group (*n* = 33). Stratified random sampling was applied in the subject selection, whereby subjects were divided into nine strata based on the district of their preschool premises: i) Gombak, ii) Hulu Selangor, iii) Hulu Langat, iv) Klang, v) Kuala Langat, vi) Kuala Selangor, vii) Petaling, viii) Sabak Bernam and ix) Sepang. A total of three to four subjects were selected from each stratum based on random numbers generated from Statistical Package for the Social Sciences (SPSS) version 23.0.

After identification, the selected preschool teachers were briefed on the study and those who consented signed the study consent form. For the preschool children involved in the study, informed consent was collected from their parents and only those whose parents allowed their children to participate in the study were included for screening.

### Recertification Training Module for Preschool Vision Screening

Three sessions of focus group discussions were conducted before drafting the recertification module for preschool vision screening to obtain insights and feedback from the preschool teachers involved in the earlier training module. A draft of the recertification module was then produced containing the outcome of the focus group discussions and then further refined by preschool vision screening stakeholders, including subject matter experts in optometry, early childhood education, public health services, preschool teaching and preschool administration in the refinement workshop. The recertification module of preschool vision screening then underwent a pilot study and where it achieved a factor loading of > 0.75 for each of the items in a confirmatory factor analysis with a highly significant (*P* < 0.001) positive relationship between Factors I, II and III in the Spearman’s rank correlation test performed.

This recertification training module for preschool vision screening used the KieVision^™^ Preschool Vision Screening Kit as the vision screening tool for preschool teachers. The recertification training module for preschool vision screening was divided into two sessions: i) revision and ii) case scenario discussion. In the revision session, teachers in the Study Group received 1-h refresher modules on four major topics: i) Introduction to vision screening, ii) Understanding your eyes’ structure, iii) Normal and abnormal vision, and iv) Steps to conduct preschool vision screening. Then, a 2-h case scenario discussion was conducted for the Study Group members to discuss the difficulties and challenges that preschool teachers encounter during vision screening tests, how to conduct vision screening tests on special children and a knowledge consolidation session through case studies and case referral. Meanwhile, the Control Group was briefed for 30 min on the step-by-step procedures to carry out the preschool vision screening without a case discussion session.

### The Conduct of Vision Screening on Preschool Children

Both groups of preschool teachers were then required to conduct a vision screening programme on 15 preschool children aged between 4 years old and 6 years old at their respective preschool premises. On the preschool children, the retrained screeners and optometrists performed four vision screening tests: i) observation of the child’s behaviour, ii) observation of the external eye, iii) Hirschberg test and iv) distant VA test. Optometrists would then repeat the screening protocol on the same preschool children 2 weeks after the teachers had completed their screening session. Preschool children who failed the vision screening tests performed by the preschool teachers, optometrists or both were then referred to the Optometry Clinic, Universiti Kebangsaan Malaysia for comprehensive eye examination and management.

### Data Analysis

The study results were analysed using the Statistical Package for the Social Sciences (SPSS) version 23.0. The mean, standard deviation, range and percentage of the screening test results performed by the preschool teachers in both groups and the optometrists were determined using descriptive analysis. The Shapiro-Wilk test was used normality test as the sample size was less than 100. A *P*-value of < 0.05 was considered statistically significant. The results of the screening test performed by the Study Group and Control Group were compared with the results of screening tests performed by optometrists. A 2 × 2 table was then constructed to determine the validity value of each test which included the sensitivity, specificity, positive predictive value (PPV) and negative predictive value (NPV) according to the method of Armitage et al. (16).

## Results

A total of 59 subjects were involved in this study: the Study Group (*n* = 31) and the Control Group (*n* = 28). Two subjects (6.1%) dropped out of the Study Group due to a medical reason and five subjects (15%) dropped out of the Control Group due to a medical reason or miscommunication on the training date. All of the subjects were females and serving in the preschools from nine districts in Selangor: i) Gombak (*n* = 6), ii) Hulu Selangor (*n* = 3), iii) Hulu Langat (*n* = 11), iv) Klang (*n* = 8), v) Kuala Langat (*n* = 3), vi) Kuala Selangor (*n* = 6), vii) Petaling (*n* = 14), viii) Sabak Bernam (*n* = 5) and ix) Sepang (*n* = 3). There was a difference between numbers in each district over time as teachers were relocated to districts. Hence, there was a variation in the number of subjects in each stratum from the earlier assignment (three to four per strata for each group). The recertification training for both groups was conducted at the Institut Kemajuan Desa (INFRA), Bangi, Selangor. The mean age of preschool teachers in the Study and Control groups was 38.10 (standard deviation [SD] = 9.26) years old and 37.61 (SD = 8.14) years old, respectively, while the mean duration of their teaching experience was 13.19 (SD = 8.58) years and 12.75 (SD = 7.14) years, respectively. The Shapiro-Wilk test was conducted on the age and teaching experience data; both were found not normally distributed (*P* < 0.001). Results from the Mann-Whitney U test showed no significant difference between the Study Group and Control Group regarding age (*P* = 0.87) and teaching experience (*P* = 0.78), respectively.

**Figure 1:**
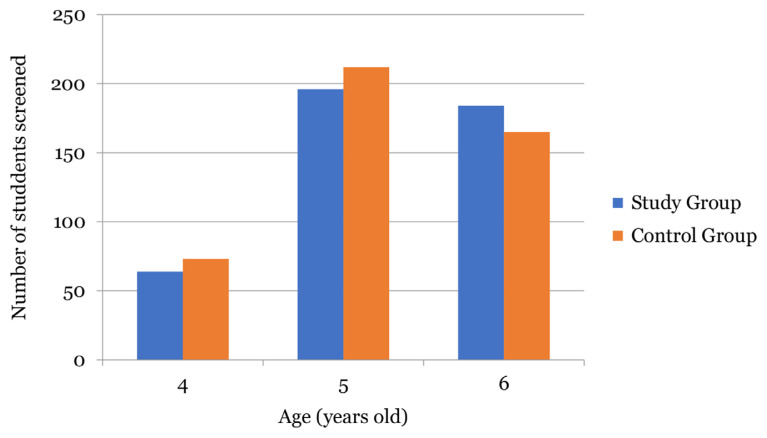
The number of children screened by preschool teachers by age in the Study Group (*n* = 444) and Control Group (*n* = 450)

A total of 894 preschool children were screened by the preschool teachers and optometrists, where the Study Group screened 444 children and the Control Group screened 450 children. The screened children comprised 427 (47.8%) boys and 467 (52.2%) girls. The age distribution of these screened children was 135 (15.0%) 4-year-olds, 411 (46.0%) 5-year-olds and 348 (39.0%) 6-year-olds. Figure 1 illustrates the number of children screened by preschool teachers by age in the Study and Control Groups.

Of the children screened by preschool teachers in the Study Group, 52 (11.7%) needed a referral for follow-up examination, while 48 (10.8%) needed a referral when screened by optometrists. Thirty-two of these children were found to have been similarly referred by both preschool teachers and optometrists. While in the Control Group, preschool teachers referred 44 (9.8%) and optometrists referred 50 (11.1%) children, while only 18 children were referred by both the preschool teachers and optometrists. Tables 1 and 2 show, respectively, the summary of the number of children referred by the Study and Control Groups and the optometrists. The validity of the screening test results performed by preschool teachers was determined according to their sensitivity, specificity, PPV and NPV, as shown in Table 3. The sensitivity and PPV of screening by the preschool teachers in the Control Group were relatively low (36.0 % and 40.9%, respectively) compared to the preschool teachers in the Study Group (66.7% and 61.5%, respectively). The analysis using the McNemar’s test showed no significant difference in the vision screening results between optometrists and both the groups, namely Study Group values (χ^2^ = 0.25, *P* = 0.62) and Control Group values (χ^2^ = 0.43, *P* = 0.51), respectively (Tables 1 and 2).

## Discussion

The descriptive analysis showed that the subjects from both groups displayed similar demographic characteristics, encompassing age, gender and teaching experience. There was also no significant difference (*P* > 0.05) in age and teaching experience between the Study and Control Groups. Of the 48 children who failed the vision screening test done by optometrists, 32 were the same children whom the Study Group referred. However, there were 16 children (33.3%) who failed the test but were ‘passed’ by the Study Group (i.e. false negatives). Therefore, the sensitivity value of the Study Group was 66.67%. These findings were similar to the studies conducted by Uddin (17) and Omar et al. (6), where they found sensitivity scores of 68.0% and 67.7%, respectively. In the Control Group, only 18 of 50 children failed vision screening, but when reviewed by optometrists, 32 children were identified (64.0%) instead. Hence, the sensitivity value for the Control Group was 36.0%, which was almost two times less sensitive in identifying vision impairment among preschool children. This study found that preschool teachers of the Study Group had better ability and accuracy in detecting visual impairment in preschool children through the vision screening programme.

The Control Group preschool teachers’ performance in detecting visual impairment showed a similar trend to that of a previous study by Omar et al. (6), which found a sensitivity value of 26.7% in identifying visual impairment by teachers who had never been given any training in performing a preschool vision screening test. These findings suggest that recertification is an important step to ensure that trained preschool teachers vision screeners can maintain their ability to conduct vision screening competently. The PPV in the Control Group was found to be lower, i.e. 40.9% when compared to the findings of the Study Group (61.5%). The PPV values of the Study Group were comparable to the findings of earlier studies, which had reviewed the conduct of training for vision screening to teachers (6, 17, 18). There was a probability of 59.1% for the Control Group preschool teachers to make over-referrals due to the high rate of false positives. Whereas in the Study Group, over-referral only occurs in about one-third of the referral cases by preschool teachers. These findings indicate that without retraining, the preschool teachers may lose their ability to perform visual screening.

In this study, we found differences in the competency levels between the Study and Control Groups. The possible explanations include that the Study Group preschool teachers completed a recertification course, where retraining on the theory and performing practical training on vision screening were provided. Besides, there was also a session concerning case scenarios where discussions and opportunities to ask questions about challenges, obstacles and doubts they encountered when conducting vision screening tests. We found that the preschool teachers from the Study Group demonstrated good competency in detecting preschool children with abnormal behaviour, abnormalities of the external eye and children with strabismus. Varying VA findings were used during the recertification training as case studies for the preschool teachers to practice on and identify the VA value examples which would make the tested children either pass or fail based on the normative/standard values from the preschool children’s age. These exercises provide a better understanding and greater confidence to the preschool teachers. Besides, these exercises also strengthen the trained preschool teachers’ competence when conducting preschool children’s vision screening, leading to more effective and valid vision screening programme results.

In this study, we also noted that preschool teachers from the Study and Control Groups exhibited a very satisfactory specificity value with NPV values of at least 92%. These findings indicate that preschool teachers could distinguish children who were free from any visual impairment. Previous studies by Chui et al. (19), Khandekar (20), Bušić et al. (21), Uddin (17) and Omar et al. (6) support these findings as well. However, the sensitivity and PPV values of the Control Group were lower when compared to the Study Group, i.e. the same as found with preschool teachers who never trained to perform vision screening tests (6, 22). Hence, it can be summarised that preschool teachers who had undergone recertification training courses could maintain competency in detecting children’s vision impairment. The level of specificity and sensitivity in the Study Group was similar to that in a previous study by Uddin (17) and Omar et al. (6), which concerned preschool teachers who had received initial preschool vision screening training only. These findings were particularly seen in preschool teachers who attended recertification training and demonstrated competence in performing vision screening tests, particularly those that involved complex concepts, namely the Hirschberg’s test and the distance VA test using Lea^™^ symbol charts.

Additionally, from a total of 894 preschool children screened, 98 preschool children failed the vision screening test when performed by optometrists. These findings suggest that the prevalence of visual impairment among preschool children was 11.0%, of which 95.9% of the referred children failed distance VA testing. The prevalence of visual impairment reported in our study was consistent with that of Chew et al. (23), whose findings were determined in the state of Johor, Malaysia (12.5%) but was higher when compared to the studies by Duratul Ain et al. (24) and Premsenthil et al. (25), which reported findings of 5% and 6.7%, respectively. The study is consistent with the predictions of Varma et al. (22) regarding an increased trend in the prevalence of visual impairment, as observed in the study. Thus, these findings illustrate the importance of expanding the vision screening test programme for preschool children at the national level to enable early detection and therefore allow treatment to commence as soon as possible.

The main limitation of this study is the lack of an optometrist to perform vision screening tests for preschool children at the preschool premises. As mentioned, the optometrist performed a vision screening test on the same preschool children 2 weeks after the preschool teachers performed the same initial screening test. Whereas, with only one optometrist carrying out these tests on 894 children in a short period is considered a very urgent task. Furthermore, only some children who must be screened attended the class on the day of the optometrist’s visit. Therefore, the optometrist visited some of these preschool premises three times. However, this has avoided inter-observer bias. In addition, the effect of intra-observer bias was also not determined or controlled in the pilot study. Although optometrists are usually considered certified vision screeners, it is suggested that confounding factors like this need to be considered before proceeding to the next phase of the study so that the research results are more accurate.

## Conclusion

In conclusion, the recertification training module developed in this study has successfully supported preschool teachers in maintaining their competency to conduct preschool vision screening using KieVision^™^ Preschool Vision Screening Kit. The increase in the level of knowledge and retention of skills in the preschool teachers who attended the recertification training demonstrates the effectiveness of this module.

## Figures and Tables

**Table 1:** Results of preschool vision screening by preschool teachers in the Study Group (*n* = 31) and optometrists

Variable		Optometrists			χ^2^ statistic^a^ (df)	*P*-value^a^

		Referred, *n* (%)	Not referred, *n* (%)	*n*		
Study Group	Referred	32 (7.2)	20 (4.5)	52	0.25 (1)	0.62
	Not referred	16 (3.6)	376 (84.7)	392		

Note:

a McNemar’s chi-squared test with continuity correction

**Table 2:** Results of preschool vision screening by preschool teachers in the Control Group (*n* = 28) and optometrists

Variable		Optometrists			χ^2^ statistic^a^ (df)	*P*-value^a^

		Referred, *n* (%)	Not referred, *n* (%)	*n*		
Study Group	Referred	18 (4.0)	26 (5.8)	44	0.43 (1)	0.51
	Not referred	32 (7.1)	374 (83.1)	406		

Note:

a McNemar’s chi-squared test with continuity correction

**Table 3:** Validity values of preschool vision screening by preschool teachers in the Study Group (*n* = 31) and Control Group (*n* = 28)

Validity	Study Group	Control Group
Sensitivity	66.7%	36.0%
Specificity	95.0%	93.5%
PPV^b^	61.5%	40.9%
NPV^c^	96.0%	92.1%

Notes:

b positive predictive value;

c negative predictive value
